# Biological Matrix‐Assisted Apexification: A Case Report Using PRF and MTA in Immature Necrotic Teeth

**DOI:** 10.1155/crid/4060142

**Published:** 2026-01-22

**Authors:** Shwetana Kurundkar, Adityasingh Patel, Manoj Chandak, Savadamoorthi Kamatchi Subramani, Bernice Thomas, Anuja Ikhar, Pratik Rathod, Apurva Wamane, Priyanka Bhojwani, Apurva Deshpande

**Affiliations:** ^1^ Department of Conservative Dentistry and Endodontics, Sharad Pawar Dental College and Hospital, Wardha, Maharashtra, India, dmimsu.edu.in; ^2^ Department of Restorative Dental Science, College of Dentistry, Jazan University, Jazan, Saudi Arabia, jazanu.edu.sa; ^3^ Mediclinic Welcare Wellness Center, Mediclinic Welcare Hospital, Dubai, UAE

## Abstract

It is challenging to treat an infected nonvital young permanent tooth endodontically because there is a lack of natural apical constriction. In order for the obturating material to be condensed, an apical barrier must be induced. Calcium hydroxide has historically been used to induce apexification. However, due to prolonged treatment time, multiple visits, and unpredictability in barrier formation, newer bioactive materials such as mineral trioxide aggregate (MTA) have been introduced for faster and more predictable outcomes. This case report presents the use of platelet‐rich fibrin (PRF) as a biological internal matrix in conjunction with MTA for apexification of immature necrotic teeth. Although a single‐visit procedure was initially planned, the presence of persistent exudate necessitated a multivisit approach to ensure thorough disinfection before final obturation.

## 1. Introduction

A young permanent tooth with pulp necrosis cannot achieve apical closure due to the cessation of root development. The root walls of aforementioned teeth are fragile and thin, without a closed apex. A tooth with an immature apex is difficult to treat endodontically because the root canal system cannot be completely cleaned, disinfected, and sealed. Nonvital teeth with an immature apex were traditionally treated by leaving the root canal untreated, along with mechanical instrumentation, customized filling of the material, paste fill, short fill, and apical surgery [1]. As a consequence of restricted success experienced with these techniques, much attention was given to the phenomenon of apexogenesis or apexification [2].

The process of apexification is a try to instigate apical closure in nonvital teeth with incomplete roots (immature apex) through formation of mineralized tissue in the apical region. Apexification has been performed with a variety of materials such as hydroxides of calcium or barium, zinc oxide, magnesium oxide, tricalcium phosphate, and calcium phosphate collagen gel. With a history of long‐term clinical success, calcium hydroxide is the most commonly recommended apexification material [3–5]. There are several disadvantages of calcium hydroxide, such as prolonged duration of treatment time, multiple visits and radiographs, difficult recall management, and increased risk for root fracture post calcium hydroxide dressing for extended periods [6].

Mineral trioxide aggregate (MTA) is an artificial barrier which can be utilized as a substitute for calcium hydroxide apexification. Employment of MTA for apexification has yielded consistently positive results in the literature. MTA apexification offers the following advantages: (i) treatment time is reduced, (ii) no alterations in the dentine’s mechanical properties, (iii) minimal delay in restoring the tooth, (iv) stimulation for repair, and (v) exceptional biocompatibility [7]. Overfilling and underfilling are two technical challenges associated with MTA as an apical barrier. In 1992, Lemon coined the “internal matrix concept” to treat perforations of the root. He suggested that perforation repair material was condensed against an external barrier and matrix formed by placing hydroxyapatite through the perforation [8]. Later, the modified internal matrix concept was given by Bargholz in 2005, which suggested use of collagen as a completely resorbable barrier matrix along with MTA as a repair material for perforation [9]. In order to repair perforations, a matrix is required to control the repair material. A similar idea is used in placing MTA apical barriers in immature teeth.

The second‐generation platelet concentrates, that is, platelet‐rich fibrin (PRF), are a resorbable matrix material. This can serve as an internal matrix that the MTA plug can be positioned against. Firstly, Choukroun et al. introduced PRF. In addition to being easy to prepare and autologous, it promotes wound healing, bone growth, regeneration and maturation of bone, and hemostasis [10]. PRF also serves as a biologically active scaffold rich in growth factors such as TGF‐*β*, PDGF, and VEGF, which enhance tissue healing and cellular regeneration. When used in conjunction with MTA, it provides a stable internal matrix that allows better placement control and improved apical sealing.

Recent advances have also led to the development of newer bioceramic materials derived from conventional MTA formulations, such as MTA Repair HP and Bio‐C Repair (Angelus, Londrina, Brazil), which demonstrate enhanced handling, improved radiopacity, and reduced tooth discoloration due to the replacement of bismuth oxide with alternative radiopacifiers [11]. However, despite these innovations, conventional MTA remains widely used due to its extensive clinical validation and predictable biological response [12].

The present case report describes the management of an immature necrotic tooth using MTA in combination with PRF, representing a biological matrix–assisted approach to apexification. The incorporation of PRF not only facilitated precise MTA placement but also promoted faster and more organized periapical healing [13].

## 2. Case Presentation

**Figure 1:**
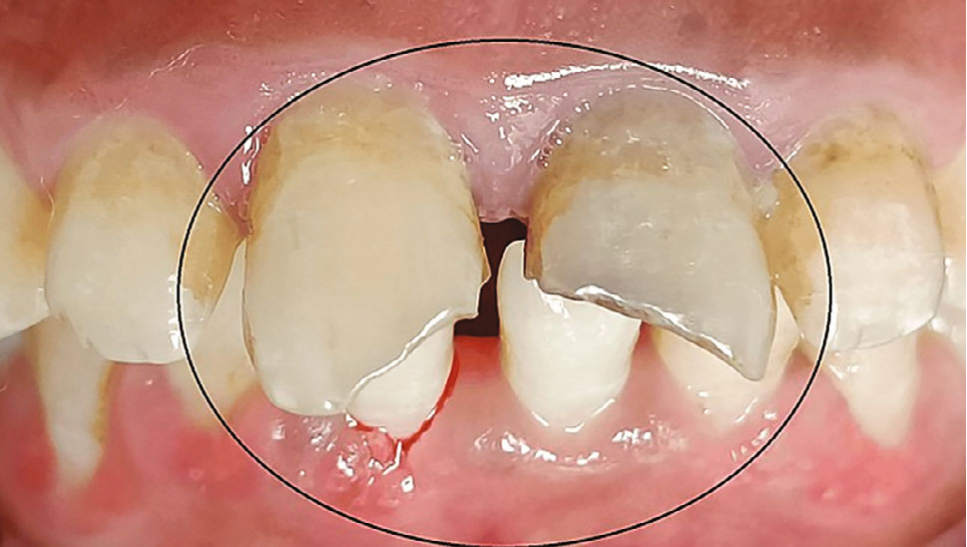
Preoperative clinical image.

A female patient of 16 years reported to the outpatient department complaining about the discoloration in the upper anterior teeth. On intraoral examination, Tooth Number 11 and 21 had an Ellis Class IV fracture (Figure 1).

**Figure 2:**
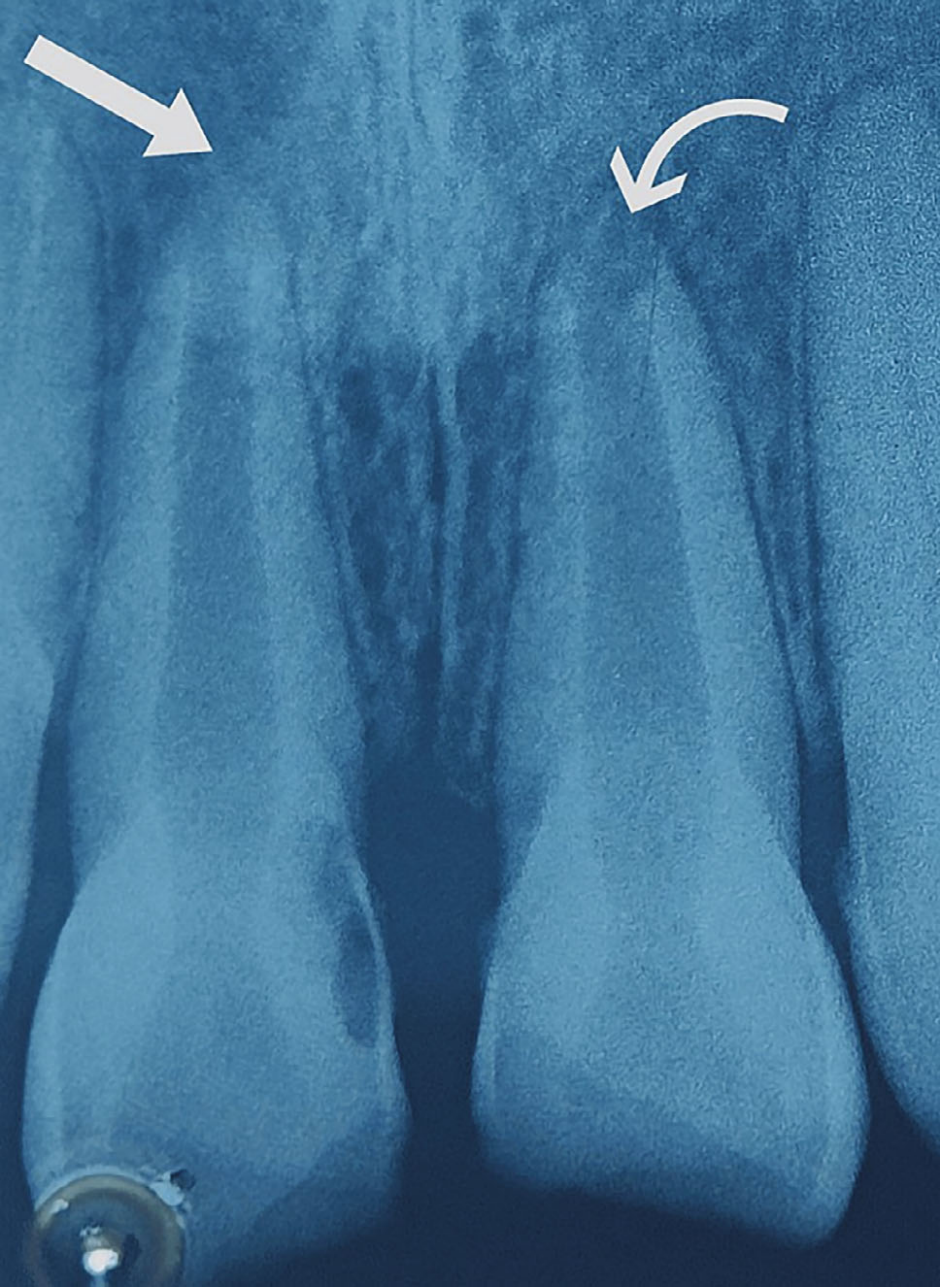
Preoperative radiograph with 11 and 21. The arrows are drawing attention to the open apex.

On vitality testing, the teeth did not show response to cold test, heat test, and electric pulp test. The intraoral periapical radiograph with the Tooth Number 11 and 21 showed an open apex with an associated periapical lesion (Figure *2*).

**Figure 3:**
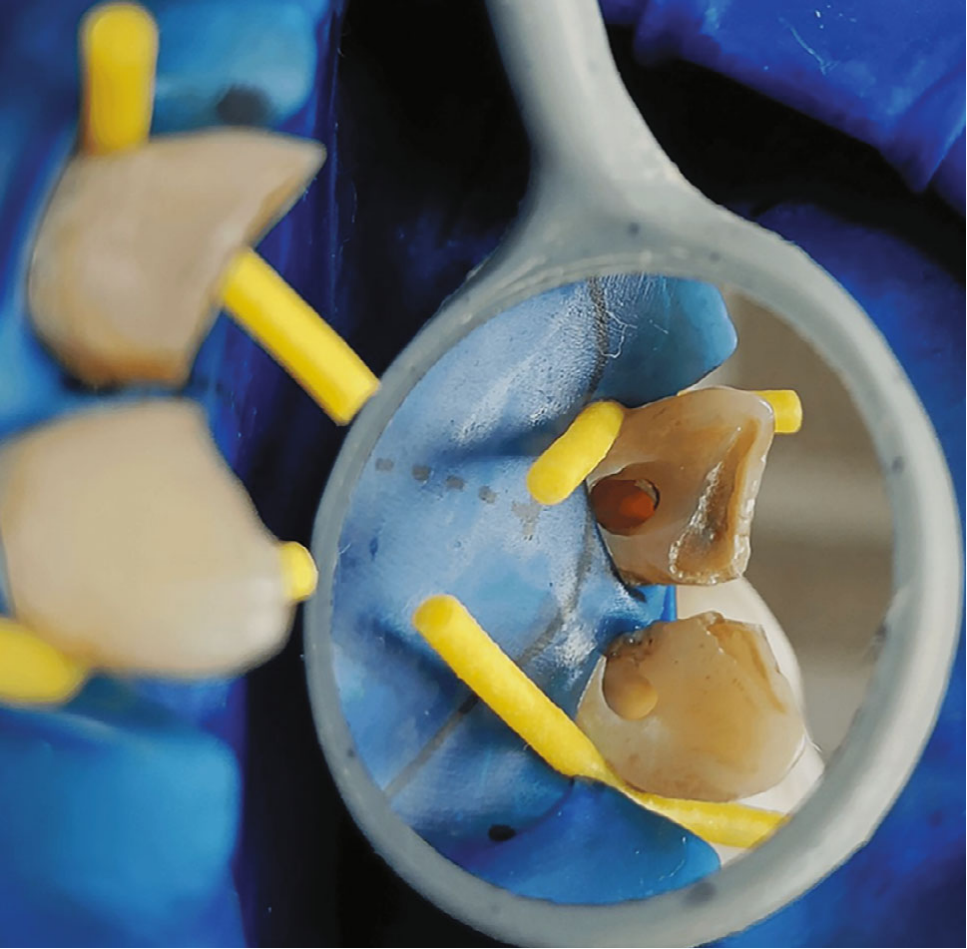
Access cavity opening with 11 and 21.

The recommended treatment plan involved endodontic therapy with a single‐visit apexification procedure using MTA as an apical barrier. The local anesthesia was administered, followed by rubber dam placement. The access opening was done with 11 and 21 (Figure 3).

Working length determination was done using No. 20 K file using an apex locator (Root ZX, Morita, Tokyo, Japan), and then reassured by taking a radiograph. The biomechanical preparation was executed with a No. 80 K file (Dentsply Maillefer, Ballaigues, Switzerland) using circumferential filing motion. Simultaneous irrigation with 1% NaOCl (sodium hypochlorite) and saline was used for root canal debridement, and 2% chlorhexidine (CHX) was utilized as final irrigant. By using a paper point, the canal drying was done, subsequently the dressing of triple antibiotic paste (ciprofloxacin, metronidazole, and minocycline) given as an intracanal medicament. However, during this appointment, persistent exudation from the canal was observed, indicating incomplete disinfection. Therefore, the treatment plan was modified from single‐visit to multivisit apexification to ensure complete canal sterilization before obturation.

The intracanal medication has been changed at the 1‐week recall appointment. Following this, during the 2‐week recall appointment, calcium hydroxide and propylene glycol were given as an intracanal dressing. This combination was used to allow sustained release of calcium and hydroxyl ions for extended antimicrobial activity and neutralization of residual infection. The tooth was nontender on percussion and asymptomatic during the 5‐week follow‐up.

After confirming canal dryness and absence of exudation, the use of a PRF membrane was planned as an internal biological matrix, along with MTA as the apical barrier. Saline irrigation was done extensively to remove traces of calcium hydroxide, followed by further chemomechanical debridement using NaOCl and saline, and final irrigation with 2% CHX.

**Figure 4:**
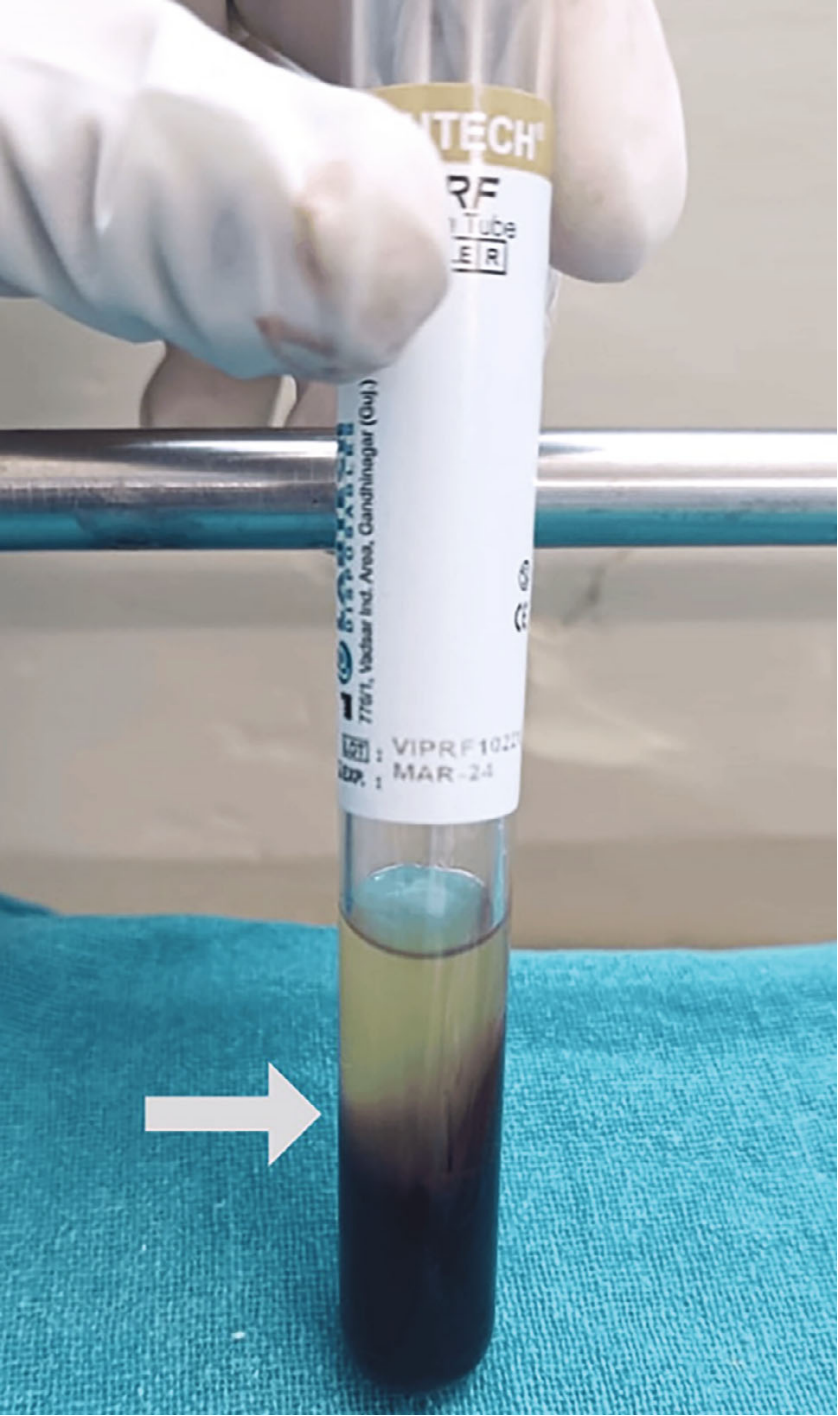
PRF in sterile test tube.

Following the protocol outlined by Dohan et al., a PRF membrane was prepared. A volume of 8.5 mL of blood was drawn by venipuncture from the antecubital vein and centrifuged for 10 min at 3000 rpm using a tabletop centrifuge (REMI R8C, Mumbai, India). Upon centrifugation, three layers were obtained—acellular platelet‐poor plasma on the top, PRF clot in the middle, and red blood cells at the bottom (Figure 4).

**Figure 5:**
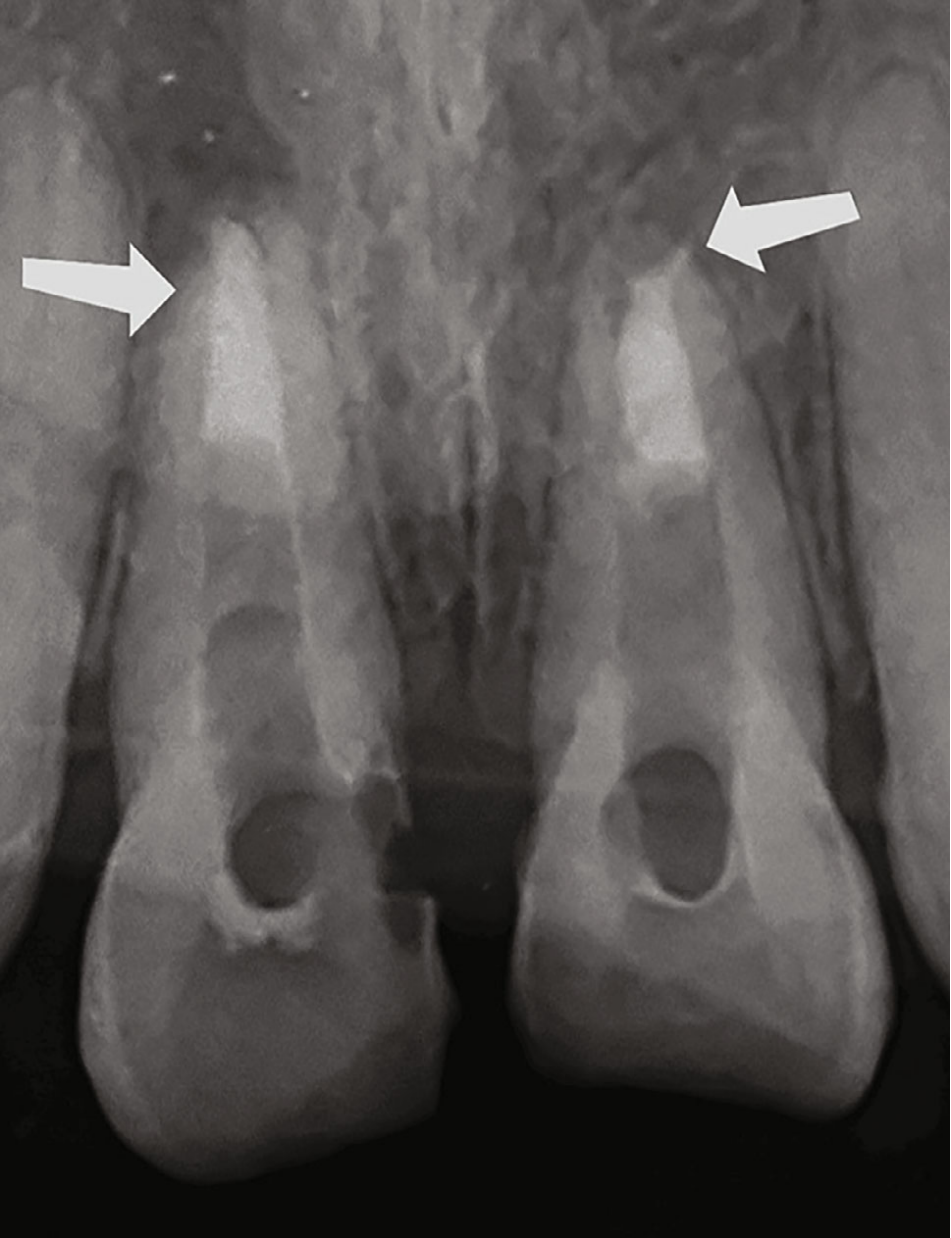
Apical barrier of MTA with 11 and 21. The arrows are drawing attention to the MTA plug which is acting as apical barrier.

The PRF clot was gently compressed in sterile gauze to obtain a membrane, which was then sectioned into two halves. Under an operating microscope, the PRF membrane was carefully inserted into the canal and compacted using stainless steel hand pluggers (Dentsply Maillefer, Ballaigues, Switzerland) to form an apical barrier. Following the manufacturer’s instructions, MTA (Angelus, Londrina, Brazil) was mixed and condensed against the PRF matrix in the apical third of the canal. Hand pluggers were used to compact successive increments of MTA until a 5‐mm apical plug was formed (Figure 5).

A moist cotton pellet was placed over the MTA, and the access cavity was sealed with a temporary restorative material. The 1‐week interval allowed adequate setting of MTA and ensured dimensional stability before final obturation.

**Figure 6:**
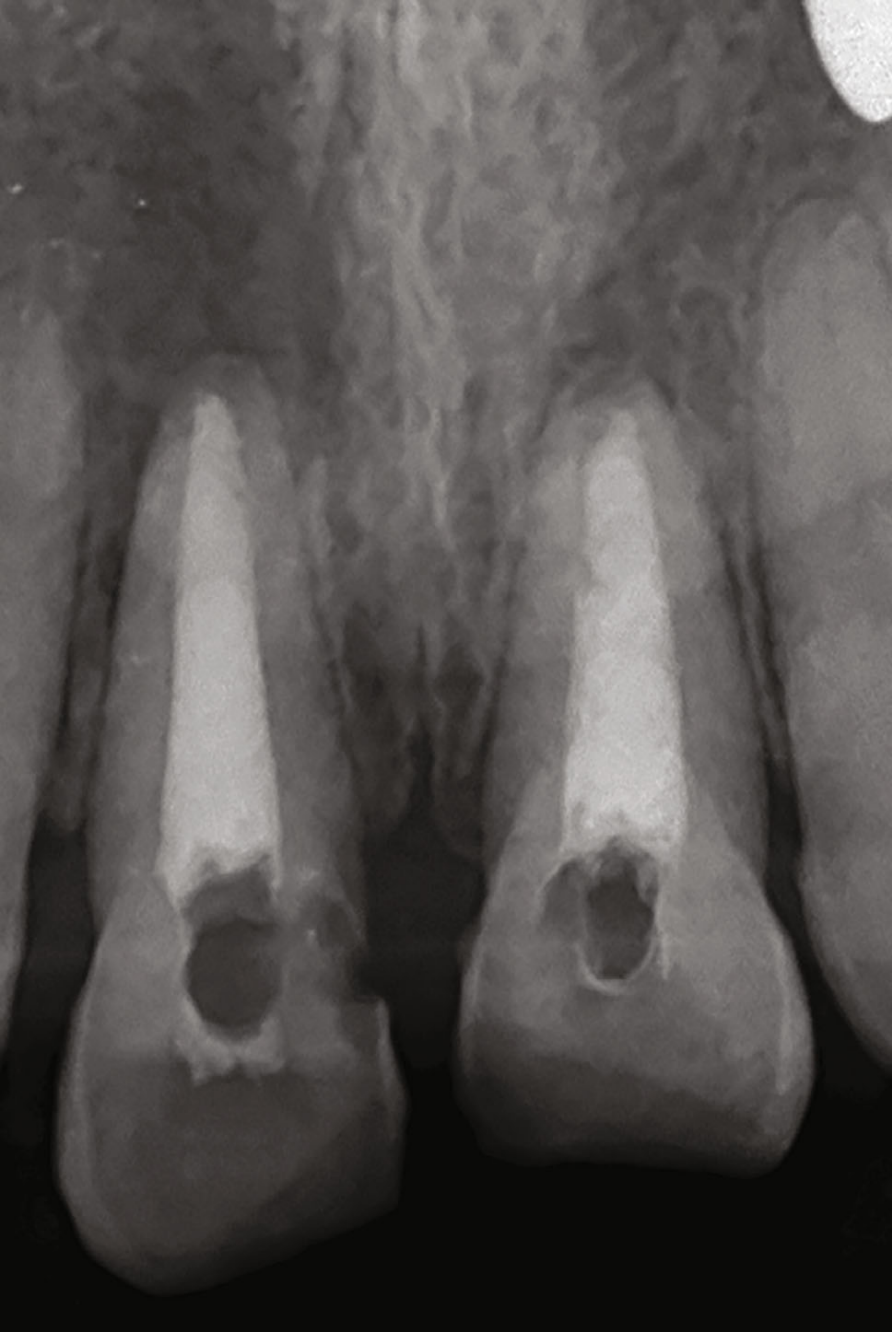
Backfill with thermoplastized obturation 11 and 21.

At the next visit, the patient was asymptomatic. After rubber dam isolation, the temporary restoration and cotton pellet were removed, and MTA set was verified by gentle probing. The canal was obturated using injectable thermoplasticized gutta‐percha (Obtura II, Obtura Spartan, United States) and AH Plus sealer (Dentsply DeTrey, Konstanz, Germany) (Figure 6).

**Figure 7:**
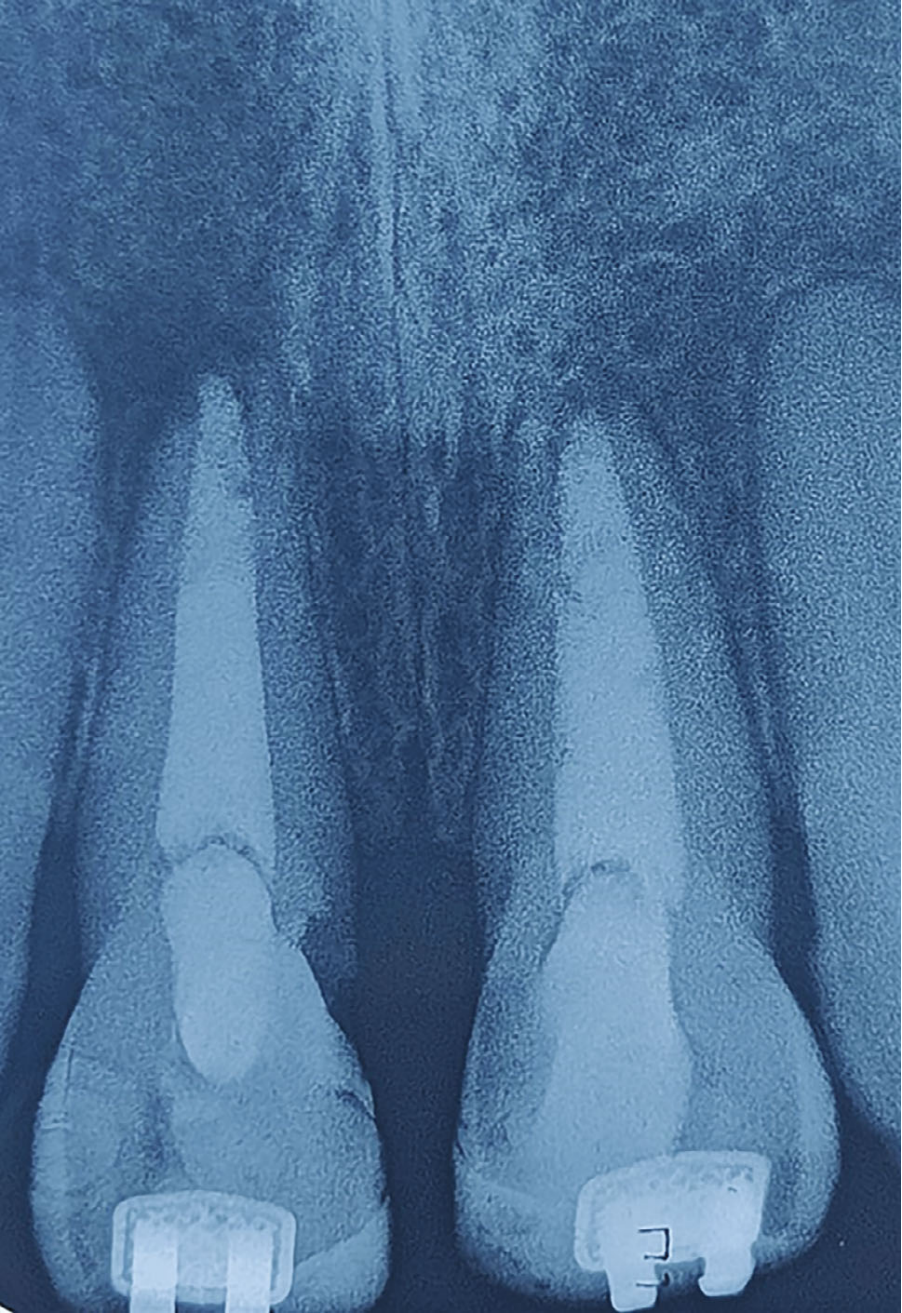
One year recall radiograph with 11 21.

The access cavity was restored using direct composite resin (Filtek Z250, 3M ESPE, United States). Before final polishing, in‐office bleaching using 35% hydrogen peroxide (Opalescence, Ultradent, United States) was performed to correct coronal discoloration and improve esthetics. The patient was recalled after 1 year for clinical and radiographic evaluation. The follow‐up periapical radiograph showed a reduction in the size of the periapical radiolucency, indicating healing and bone formation (Figure 7).

The clinical and radiographic findings demonstrated successful apical barrier formation and periapical healing, confirming the effectiveness of the PRF‐assisted MTA apexification technique.

## 3. Discussion

The objective of an apexification technique is to create an apical seal for prevention of the toxins and bacteria of the pulp canal from entering into periradicular space. To prevent an overfill of the root canal, this barrier provides condensation of the retrograde filling material and confines the endodontic filling material into the canal [14, 15]. Historically, calcium hydroxide was one of the best materials for apexification. Based on previous studies, this technique has been shown to be 100% effective, taking 12–19 months on average for the barrier to formation [11]. With such a long‐term, multiple‐appointment procedure, patient compliance is difficult; moreover, prolonged exposure to calcium hydroxide affects the mechanical properties of root dentine, causing it to be more prone to fracture [12]. Due to this, apexification in a single visit has been advised. There are several materials used for apexification, which are calcium hydroxide, tricalcium phosphate, freeze‐dried dentine, freeze‐dried bone, proplast and collagen calcium phosphate [13, 16–20]. Currently, for single‐visit apexification, MTA is a preferred material due to the excellent biocompatibility, minimal microleakage, and ability to promote the formation of periodontium and bone.

However, in the present case, although single‐visit apexification was initially planned, persistent intracanal exudate following debridement necessitated modification to a multivisit protocol. This approach allowed extended disinfection and better control of infection before final obturation. Triple antibiotic paste was used initially for 1 week to reduce microbial load, followed by calcium hydroxide mixed with propylene glycol for 4 weeks to ensure sustained antimicrobial action. This staged approach has been reported to enhance canal sterilization and improve prognosis in cases with persistent exudation.

Creating an artificial barrier at the root end presents the problem of limiting the obturating material to the root tip to prevent overextrusion [21]. Utilizing a PRF substance will limit the root end material at the root tip and prevent material from pushing out into the periodontium. Different matrix substances that could be utilized are resorbable collagen, calcium hydroxide, calcium sulfate, and hydroxyapatite [9, 22, 23]. A matrix has been created using PRF, which is an immune platelet concentrate.

In this case, PRF was used as a biological internal matrix against which MTA was compacted. This biological matrix offered a controlled platform for condensation, preventing overfill and improving adaptation of MTA at the apex. The PRF membrane not only served as a physical barrier but also contributed to accelerated periapical healing due to the release of autologous growth factors [24, 25].

The choice of MTA (Angelus, Londrina, Brazil) was based on its proven clinical performance and bioactivity. Although newer calcium silicate–based cements such as MTA Repair HP and Bio‐C Repair (Angelus, Brazil) offer enhanced handling, reduced discoloration, and improved radiopacity [11, 12], conventional MTA remains a reliable and well‐documented option for apexification. In this report, manual compaction of MTA was preferred instead of ultrasonic activation to avoid disturbing the PRF matrix; however, literature supports that ultrasonic activation can improve material adaptation to dentinal walls [14, 15].

An immature tooth with thin canal walls cannot be instrumented properly, so root canal cleaning and disinfection are based on chemical actions of irrigants and intracanal medications [24]. A combination of NaOCl and CHX was used in the present case to disinfect the canals. It is well known that NaOCl is toxic at high concentrations. Immature teeth with open apices often push irrigant beyond the apex, so a concentration of 1% NaOCl was used in this case. Calcium hydroxide and triple antibiotic paste were used as intracanal medications to disinfect the canals further. For a duration of 3 weeks, the calcium hydroxide was placed in propylene glycol, allowing slow release of ions and a longer duration of antimicrobial effect [25].

Radiographically, follow‐up at 1 year demonstrated significant reduction of periapical radiolucency, indicating bone regeneration and successful apexification. The combination of PRF and MTA not only achieved a hermetic apical seal but also promoted biological healing in the periradicular area.

## 4. Conclusions

The combined use of PRF and MTA offers a biologically driven and clinically predictable method for managing immature necrotic permanent teeth. PRF serves as an ideal internal matrix that supports controlled MTA condensation, provides a biological scaffold rich in growth factors, and enhances periapical tissue regeneration. MTA, in turn, establishes a durable apical barrier with excellent sealing ability and bioactivity. This case demonstrated that the PRF‐assisted MTA apexification technique effectively promotes apical closure and periapical healing even in cases requiring multivisit disinfection. The 1‐year follow‐up confirmed radiographic evidence of bone regeneration and functional recovery of the treated teeth. Thus, the PRF–MTA combination can be considered a reliable, biocompatible, and minimally invasive alternative to conventional calcium hydroxide apexification, offering both structural stability and biological healing in immature nonvital teeth.

## Consent

Written informed consent was obtained from the patient for the publication of this case report and the accompanying clinical images. A copy of the written consent is available for review by the *Editor-in-Chief of Case Reports in Dentistry* upon request.

## Disclosure

All the authors approved the final approval version of the manuscript. Shwetana Kurundkar agrees to be accountable for all aspects of the work.

## Conflicts of Interest

The authors declare no conflicts of interest.

## Author Contributions

**Shwetana Kurundkar**: conception and design of the study, clinical treatment and case documentation, acquisition of clinical data and images, drafting the manuscript, and coordinating revisions. **Adityasingh Patel**: supervision of clinical procedures, guidance in study design, and critical review for important intellectual content. **Manoj Chandak**: oversight of clinical management, interpretation of radiographic and clinical findings, and substantial contributions to manuscript revision. **Savadamoorthi Kamatchi Subramani**: support in manuscript technical refinement, contribution to academic quality improvement and guidance, and critical review of the manuscript. **Bernice Thomas**: contribution to manuscript refinement, expert input in endodontic concepts, and critical revision of the article. **Anuja Ikhar**: support in methodological planning, contribution to interpretation of data, and manuscript editing and review. **Pratik Rathod**: assistance in clinical procedures, data collection, preparation of radiographs and figures, and manuscript review. **Apurva Wamane**: support in case documentation, literature review, drafting assistance, and manuscript review. **Priyanka Bhojwani**: assistance in clinical recording, literature search, and manuscript review. **Apurva Deshpande**: support in data acquisition, contribution to case documentation and figure management, and review of the manuscript.

## Funding

No funding was received for this manuscript.

## Data Availability

The data that support the findings of this study are available on request from the corresponding author. The data are not publicly available due to privacy or ethical restrictions.

## References

[1] Guerrero F. , Mendoza A. , Ribas D. , and Aspiazu K. , Apexification: A Systematic Review, Journal of Conservative Dentistry. (2018) 21, no. 5, 462–465, 10.4103/JCD.JCD_96_18, 30294103. 30294103 PMC6161512

[2] Frank A. L. , Therapy for the Divergent Pulpless Tooth by Continued Apical Formation, Journal of the American Dental Association. (1966) 72, no. 1, 87–93, 10.14219/jada.archive.1966.0017, 2-s2.0-0013870310, 5215726. 5215726

[3] Ghose L. J. , Baghdady V. S. , and Hikmat Y. M. , Apexification of Immature Apices of Pulpless Permanent Anterior Teeth With Calcium Hydroxide, Journal of Endodontics. (1987) 13, no. 6, 285–290, 10.1016/s0099-2399(87)80045-6, 2-s2.0-0023359950, 3474347. 3474347

[4] Kerekes K. , Heide S. , and Jacobsen I. , Follow-Up Examination of Endodontic Treatment in Traumatized Juvenile Incisors, Journal of Endodontics. (1980) 6, no. 5, 744–748, 10.1016/S0099-2399(80)80186-5, 2-s2.0-0019062113. 6935385

[5] Cvek M. , Prognosis of Luxated Nonvital Maxillary Incisors Treated With Calcium Hydroxide and Filled With Gutta-Percha: A Retrospective Clinical Study, Endodontics & Dental Traumatology. (1992) 8, no. 2, 45–55, 10.1111/j.1600-9657.1992.tb00228.x, 2-s2.0-0026842824, 1521505. 1521505

[6] Andreasen J. O. , Farik B. , and Munksgaard E. C. , Long-Term Calcium Hydroxide as a Root Canal Dressing May Increase Risk of Root Fracture, Dental Traumatology. (2002) 18, no. 3, 134–137, 10.1034/j.1600-9657.2002.00097.x, 2-s2.0-0036623795, 12110105. 12110105

[7] Simon S. , Rilliard F. , Berdal A. , and Machtou P. , The Use of Mineral Trioxide Aggregate in One-Visit Apexification Treatment: A Prospective Study, International Endodontic Journal. (2007) 40, no. 3, 186–197, 10.1111/j.1365-2591.2007.01214.x, 2-s2.0-33846953503, 17305695. 17305695

[8] Lemon R. R. , Nonsurgical Repair of Perforation Defects: Internal Matrix Concept, Dental Clinics of North America. (1992) 36, no. 2, 439–457, 1349289. 1349289

[9] Bargholz C. , Perforation Repair With Mineral Trioxide Aggregate: A Modified Matrix Concept, International Endodontic Journal. (2005) 38, no. 1, 59–69, 10.1111/j.1365-2591.2004.00901.x, 2-s2.0-12144275422, 15606825. 15606825

[10] Dohan D. M. , Choukroun J. , Diss A. , Dohan S. L. , Dohan A. J. J. , Mouhyi J. , and Gogly B. , Platelet-Rich Fibrin (PRF): A Second-Generation Platelet Concentrate. Part I: Technological Concepts and Evolution, Oral Surgery, Oral Medicine, Oral Pathology, Oral Radiology, and Endodontics. (2006) 101, no. 3, e37–e44, 10.1016/j.tripleo.2005.07.008, 2-s2.0-33644557480, 16504849. 16504849

[11] Camilleri J. , Characterization of Hydration Products of Mineral Trioxide Aggregate, International Endodontic Journal. (2008) 41, no. 5, 408–417, 10.1111/j.1365-2591.2007.01370.x, 2-s2.0-42149172353, 18298574. 18298574

[12] Torabinejad M. and Parirokh M. , Mineral Trioxide Aggregate: A Comprehensive Literature Review—Part III: Clinical Applications, Drawbacks, and Mechanism of Action, Journal of Endodontics. (2010) 36, no. 3, 400–413, 10.1016/j.joen.2009.09.009, 2-s2.0-76449099081. 20171353

[13] Dohan Ehrenfest D. M. , Rasmusson L. , and Albrektsson T. , Classification of Platelet Concentrates: From Pure Platelet-Rich Plasma (P-PRP) to Leucocyte- and Platelet-Rich Fibrin (L-PRF), Trends in Biotechnology. (2009) 27, no. 3, 158–167, 10.1016/j.tibtech.2008.11.009, 2-s2.0-60349120413, 19187989. 19187989

[14] Vallés M. , Mercadé M. , Duran-Sindreu F. , Bourdelande J. L. , and Roig M. , Influence of Ultrasonic Activation on the Sealing Ability of MTA and Biodentine as Root-End Filling Materials, Journal of Endodontics. (2013) 39, no. 2, 142–145, 10.1016/j.joen.2012.10.012, 2-s2.0-84870955192.

[15] Lawley G. R. , Schindler W. G. , Walker W. A.3rd, and Kolodrubetz D. , Evaluation of Ultrasonically Placed MTA and Fracture Resistance With intracanal composite resin In a model of Apexification, Journal of Endodontics. (2004) 30, no. 3, 167–172, 10.1097/00004770-200403000-00010, 2-s2.0-2342612810, 15055436. 15055436

[16] Dominguez Reyes A. , Muñoz Muñoz L. , and Aznar M. T. , Study of Calcium Hydroxide Apexification in 26 Young Permanent Incisors, Dental Traumatology. (2005) 21, no. 3, 141–145, 10.1111/j.1600-9657.2005.00289.x, 2-s2.0-19344377962, 15876324. 15876324

[17] Rafter M. , Apexification: A Review, Dental Traumatology. (2005) 21, no. 1, 1–8, 10.1111/j.1600-9657.2004.00284.x, 2-s2.0-13144275253, 15660748. 15660748

[18] Schumacher J. W. and Rutledge R. E. , An Alternative to Apexification, Journal of Endodontics. (1993) 19, no. 10, 529–531, 10.1016/S0099-2399(06)81497-4, 2-s2.0-0027674350. 8120491

[19] Coviello J. and Brilliant J. D. , A Preliminary Clinical Study on the Use of Tricalcium Phosphate as an Apical Barrier, Journal of Endodontics. (1979) 5, no. 1, 6–13, 10.1016/S0099-2399(79)80141-7, 2-s2.0-0018290055, 296242. 296242

[20] Rossmeisl R. , Reader A. , Melfi R. , and Marquard J. , A Study of Freeze-Dried (lyophilized) Cortical Bone Used as an Apical Barrier in Adult Monkey Teeth, Journal of Endodontics. (1982) 8, no. 5, 219–226, 10.1016/S0099-2399(82)80358-0, 2-s2.0-0020134784, 7050284. 7050284

[21] Nevins A. , Finkelstein F. , Laporta R. , and Borden B. G. , Induction of Hard Tissue Into Pulpless Open-Apex Teeth Using Collagen-Calcium Phosphate Gel, Journal of Endodontics. (1978) 4, no. 3, 76–81, 10.1016/S0099-2399(78)80263-5, 2-s2.0-0017939162, 98608. 98608

[22] Alhadainy H. A. , Himel V. T. , Lee W. B. , and Elbaghdady Y. M. , Use of a Hydroxylapatite-Based Material and Calcium Sulfate as Artificial Floors to Repair Furcal Perforations, Oral Surgery, Oral Medicine, Oral Pathology, Oral Radiology, and Endodontics. (1998) 86, no. 6, 723–729, 10.1016/s1079-2104(98)90211-6, 2-s2.0-0032239969, 9868732. 9868732

[23] Mesimeris V. , Sade E. , and Baer P. N. , Calcium Sulfate as a Biodegradable Barrier Membrane—A Preliminary Report on the “Surgiplast” Technique, Periodontal Clinical Investigations. (1995) 17, no. 1, 13–16, 9055695. 9055695

[24] Siqueira J. F.Jr., Guimarães-Pinto T. , and Rôças I. N. , Effects of Chemomechanical Preparation With 2.5% Sodium Hypochlorite and Intracanal Medication With Calcium Hydroxide on Cultivable Bacteria in Infected Root Canals, Journal of Endodontics. (2007) 33, no. 7, 800–805, 10.1016/j.joen.2006.11.023, 2-s2.0-34250170834, 17804315. 17804315

[25] Hülsmann M. , Heckendorff M. , and Lennon A. , Chelating Agents in Root Canal Treatment: Mode of Action and Indications for Their Use, International Endodontic Journal. (2003) 36, no. 12, 810–830, 10.1111/j.1365-2591.2003.00754.x, 2-s2.0-0348047581, 14641420. 14641420

